# Entrenched Geographical and Socioeconomic Disparities in Child Mortality: Trends in Absolute and Relative Inequalities in Cambodia

**DOI:** 10.1371/journal.pone.0109044

**Published:** 2014-10-08

**Authors:** Eliana Jimenez-Soto, Jo Durham, Andrew Hodge

**Affiliations:** The University of Queensland, School of Population Health, Brisbane, Queensland, Australia; Institute for Health & the Environment, United States of America

## Abstract

**Background:**

Cambodia has made considerable improvements in mortality rates for children under the age of five and neonates. These improvements may, however, mask considerable disparities between subnational populations. In this paper, we examine the extent of the country's child mortality inequalities.

**Methods:**

Mortality rates for children under-five and neonates were directly estimated using the 2000, 2005 and 2010 waves of the Cambodian Demographic Health Survey. Disparities were measured on both absolute and relative scales using rate differences and ratios, and where applicable, slope and relative indices of inequality by levels of rural/urban location, regions and household wealth.

**Findings:**

Since 2000, considerable reductions in under-five and to a lesser extent in neonatal mortality rates have been observed. This mortality decline has, however, been accompanied by an increase in relative inequality in both rates of child mortality for geography-related stratifying markers. For absolute inequality amongst regions, most trends are increasing, particularly for neonatal mortality, but are not statistically significant. The only exception to this general pattern is the statistically significant positive trend in absolute inequality for under-five mortality in the Coastal region. For wealth, some evidence for increases in both relative and absolute inequality for neonates is observed.

**Conclusion:**

Despite considerable gains in reducing under-five and neonatal mortality at a national level, entrenched and increased geographical and wealth-based inequality in mortality, at least on a relative scale, remain. As expected, national progress seems to be associated with the period of political and macroeconomic stability that started in the early 2000s. However, issues of quality of care and potential non-inclusive economic growth might explain remaining disparities, particularly across wealth and geography markers. A focus on further addressing key supply and demand side barriers to accessing maternal and child health care and on the social determinants of health will be essential in narrowing inequalities.

## Introduction

The Millennium Development Goal 4 (MDG 4) targets a two-thirds reduction in under-five mortality and has prompted increased international efforts to reduce child mortality and measure progress. Evidence at the national level suggests that globally under-five mortality is declining. Nevertheless, despite global gains in reducing child mortality, disparities remain between countries and within-countries [1]–[3]. Consideration of inequalities in access to health services and health outcomes has become an issue of increasing attention in the public health arena by scholars and policy makers [1], [4]. The post-MDG agenda with its focus on universal health coverage has also drawn attention to the degree of equity in the distribution of improved health outcomes [4], [5]. Most of the research to date has focussed on urban/rural disparities and socioeconomic disadvantage. There is increasing recognition, however, of the importance of other dimensions, such as ethnicity and geography in determining health outcomes [2], [6], [7].

Cambodia is a low-income country in South East Asia with a large rural population [8]. Both the demographic and epidemiological transitions are underway and the country is experiencing rapid economic growth [8]. Recent assessments show an impressive decline in the under-five mortality rate per 1,000 live births (U5MR) from approximately 124 in 2000 to 54 in 2010 [9]. The neonatal mortality rate (NMR) has also noticeably reduced between the years 2000 and 2005, from 37 to 28, but since 2005 has stagnated with a rate of 27 in 2010 [9]. According to the recent 2010 Global Burden of Disease estimates, the main causes of under-five mortality are lower respiratory infections, preterm birth complications, congenital anomalies, neonatal encephalopathy and diarrhoeal diseases [10]. Undernutrition and micronutrient deficiency are high and are the most significant risk factors for under-five mortality [10]. Most under-five deaths occur in early infancy and neonatal mortality contributes to over a third of all under-five mortality in Cambodia [11], The leading causes of neonatal mortality are preterm birth, birth asphyxia, sepsis and pneumonia [11].

Regional inequalities, differences between rural and urban populations and disparities associated with the wealth gradient have also been observed [4], [5], [9], [12]. Over the last ten years, Cambodia has prioritised addressing health disparities particularly in relation to maternal and child health. Demand-side policies and programs have focussed on improving access to health services for the poor through a number of social protection measures. These have included user fee exemptions, health equity funds, vouchers and community-based health insurance [13]–[16]. Supply–side interventions have included the training of midwives, a midwifery financial incentive scheme, banning the use of untrained traditional birth attendants, increasing coverage of reproductive and maternal health services [4], [17] and increasing immunization coverage [18].

The policy and program focus on decreasing maternal and child health disparities warrants further analysis. While research has revealed that inequity in a range of maternal and child health service use by wealth quintile has generally decreased over time [4], to date there has been no analysis of changes in equity in neonatal health mortality. To the best of our knowledge this is the first comprehensive study of levels, trends and inequalities – both absolute and relative – of under-five and neonatal mortality in Cambodia across regions, wealth and rural/urban residence markers.

## Methods

### Ethics

This study is based on the Cambodian Demographic Health Surveys (DHS) [9]. The DHS is undertaken by the National Institute of Statistics who were responsible for the management and review of the survey, with technical assistance from international advisors. Full review of this study from an institutional review board was not sought as the datasets were anonymous, with no identifiable information on the survey participants.

### Data

The data source was the DHS series conducted in Cambodia in 2000, 2005 and 2010 as part of the global MEASURE DHS. The DHS is a nationally representative household survey, with a women's module of females aged 15 to 49 years [9]. In each of the three surveys households were sampled from 14 individual provinces and five groups of provinces resulting in 19 sampling domains, stratified into urban and rural areas. Details of the sampling design are provided elsewhere [9]. All women interviewed were asked to give a detailed history of all her live births in chronological order. Information collected included: whether a birth was single or multiple; sex of the child; date of birth; survival status; age of the child on the date of interview or age at death of each live birth.

In the present study rural/urban place of residence, region and household wealth were used as stratifying variables across which disparities in child mortality were estimated. Provinces were categorised into five agro-ecological regions commonly used in socioeconomic assessments in Cambodia. These provinces were grouped into areas with broadly similar terrain, accessibility, climate and economic activity. These regions are the Plains, Tonle Sap, Plateau/Mountain, Phnom Penh (the base group) and Coastal [19]. Household wealth was gauged through an asset index, which provides a reliable proxy measure of wealth in the absence of household income or consumption that is not contained within the DHS [20], [21]. The survey-provided asset index was constructed for each survey round using principal component analysis (PCA), incorporating three categories of assets: durable consumer goods (e.g. ownership of a refrigerator, television, motorbike, etc.), quality of the dwelling (e.g. roof material, floor material etc.) and access to utilities and infrastructure (e.g. main source of drinking water, type of toilet facility). Using the factor scores from the first principal component, socioeconomic categorisation was obtained by ranking, then classifying households within the distribution into three groupings. Thus the derived indices are relative measures of socioeconomic status and not measures of absolute poverty [22], [23].

### Analysis and Measures of Disparities

The outcome measures were under-five and neonatal mortality. These rates were estimated directly following the methods of Rajaratnam and colleagues [24]. The estimation procedure is identical at both national and sub-national levels, with rates estimated biennially due to the relative rarity of child deaths. We assembled mortality records for each child detailing the life or death in each month of the first five years of each child's life, which we denote person-months data. For each two-year time period, survival rates for the following age groups were calculated: 0 to 1; 1 to 11; 12 to 23; 24 to 35; 36 to 47; and 48 to 59 months. The mortality rate is computed as one minus the survival rate. The biennial survival rates (i.e. mean survival probability) for the aforementioned age groups were directly estimated by dividing the total number of person-months where children were alive by the total number of person-months in the time period of interest, accounting for sample weights. The survival rates for each age group are then used to obtain the desired child mortality indicator by amalgamating the correct age groups: that is, under-five mortality is one minus the survival rates from all the age groups multiplied together, while neonatal mortality is one minus the survival rate from birth to 1 month. Confidence intervals were constructed using 1,000 simulations, with the survival probability generated assuming a binomial distribution and the lower and upper confidence bounds extracted from the 2.5^th^ and 97.5^th^ percentiles [24], [25]. This standard simulation method captures both sampling and model uncertainty [24], [26].

In context of declining mortality rates, it is well known that different conclusions about inequalities can be drawn depending on the scale used [27]. Hence, both absolute and relative measures of inequalities were computed, namely: rate difference (RD) and the slope index of inequality (SII) on the absolute scale and rate ratio (RR) and the relative index of inequality (RII) on the relative scale [27]–[29]. These measures were computed using the biennial mortality rates. Further details on the computation of these measures are provided in Box S1 in File S1. The main advantage of the RIIs and SIIs over the RDs and RRs is that the former accounts for changes in the distribution of the equity marker. The RII and SII are computed via weighted linear regression [30] and the need for ordinal social groups to rank the population implies that the RIIs and SIIs can only feasibly be computed using wealth. For the non-wealth equity markers, RDs and RRs are computed for each sub-population in reference to a base group, which is chosen as the group with the lowest average under-five mortality rate over the sample period. Confidence intervals for the RIIs and SIIs were calculated using standard methods outlined by Hayes and Berry [31]; and for the RDs and RRs were computed using the simulation process utilised for the mortality estimates. Comparisons over time were gauged by comparing the mortality rates and measures of inequalities over the sample period and considering 95% confidence intervals and *p*-values for tests of the statistical significance of a linear trend in these estimates [29]. In the cases of RRs and RIIs, we used the natural logarithm of these measures in the regressions. We used Newey-West standard errors (using one lag) in these regressions, which are robust to both heteroskedasticity and serial correlation.

All statistical analyses were conducted using the two software programs, *Stata* and *R*.

## Results

**Figure 1:**
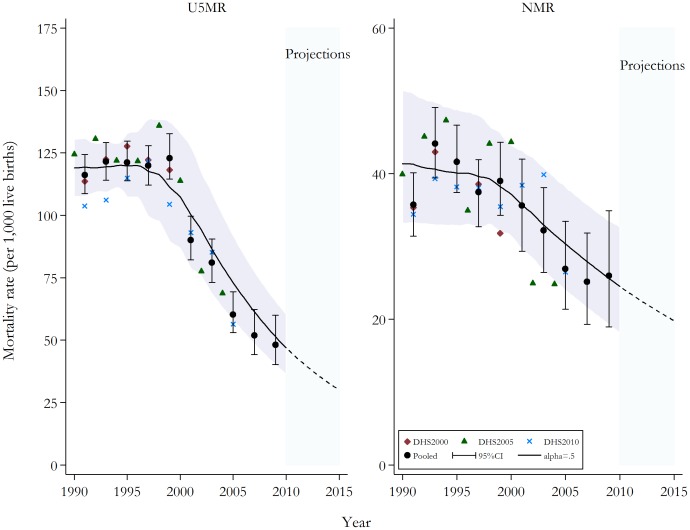
Under-five and neonatal mortality rates (per 1,000 live births) at the national level: actual 1990–2010; projected to 2015. *Notes*: National estimates by source and the pooled data are displayed. The solid represent the continuous mortality estimates calculated from the two-year estimates and loess regression with a smoothing parameter of 0.5 [43]. semi-broken lines are projections computed using the last set of parameter estimates for the Loess regression [44]. The shaded area signifies the corresponding 95% confidence intervals. U5MR, under-five mortality rate; NMR, neonatal mortality rate; DHS, Demographic Health Survey; CI, confidence intervals.

National estimates derived using the above outlined methodology confirms the general pattern reported elsewhere. Figure 1 presents national estimates. The U5MR remained constant during the 1990s, hovering around approximately 121 deaths per 1,000 live births, with 120 (95% CI 111 to 128) in 1989–90 and 123 (95% CI 115 to 133) in 1999–2000. During the 2000s, U5MR declined dramatically, with the most recent estimate suggesting mortality rates have more than halved to 48 (95% CI 40 to 60) in 2009–10. The pattern of reduction in the NMR was similar, with the exception of possible stagnation since 2005 at approximately 25 deaths per 1,000 live births. Despite these reductions at the national level, closer examination of trends in disparities showed significant within-country inequalities.

**Figure 2:**
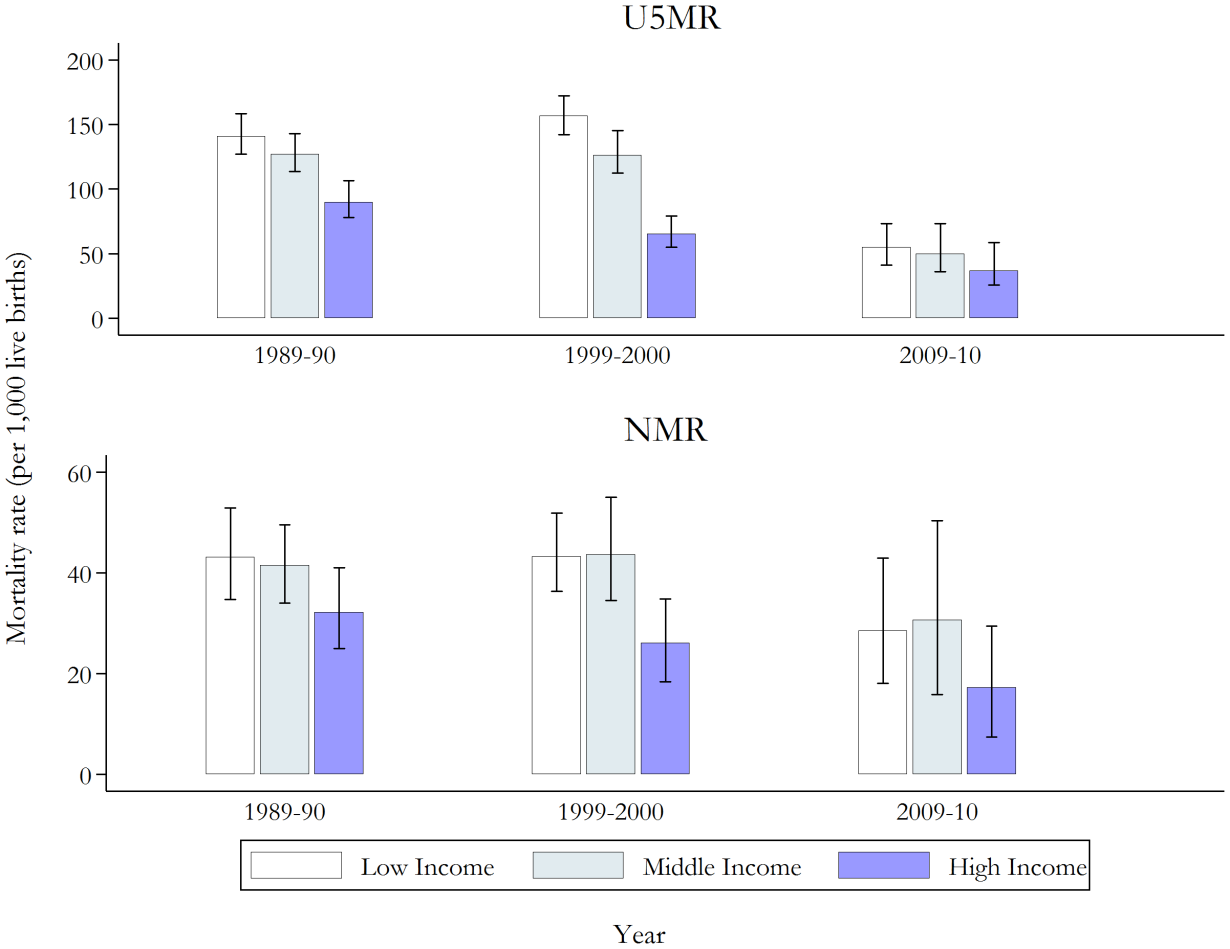
Under-five and neonatal mortality rates (per 1,000 live births) by wealth groups for selected two-year periods, with 95% confidence intervals. *Notes*: See File S1 for full results. The population was divided into thirds using the wealth index. U5MR, under-five mortality rate; NMR, neonatal mortality rate.

As to be expected, high income households experience lower rates of under-five and neonatal mortality compared to households with poorer socioeconomic status. With the observed reductions in under-five mortality rates by wealth (see Figure 2), we find the well-known pattern of reducing absolute but rising relative inequalities as reported in Table 1. However, with respect to U5MR none of the trends are statistically significant at conventional levels. The results show increasing trends in both relative and absolute inequality for neonates. For example, the RDs for neonatal mortality have increased from 10.9 (95% CI −1.6 to 23.9) in 1989–90 to 11.26 (95% CI −5.3 to 30.7) in 2009–10 for low income households and from 9.3 (95% CI −2.6 to 20.1) in 1989–90 to 13.4 (95% CI −5.3 to 33.9) in 2009–10 for middle income households. Similarly, the SII was found to have increased from 16.18 (95% CI −69.2 to 101.6) to 16.70 (95% CI −151 to 184.4) over the same period. Note, however, when different assumptions (i.e. the standard variance estimator and Huber-White sandwich estimator) with respect to the standard errors are used (results not reported), the positive trends in absolute inequalities are statistically significant only in a few cases. Positive trends in RRs and RIIs are associated with greater statistical significance than the trends in absolute inequalities, and are robust to changes in the assumptions underlying the standard errors.

**Table 1:** Inequalities in under-five and neonatal mortality by wealth for selected years, with 95% confidence intervals and *p*-values for trend.

Measure	U5MR				NMR			
	1989–90	1999–2000	2009–10	Trend	1989–90	1999–2000	2009–10	Trend
*Relative Inequalities*								
RR								
Low income	1.57	2.40	1.50	1.037	1.34	1.65	1.65	1.070
	(1.29 to 1.91)	(1.93 to 2.93)	(0.87 to 2.39)	[0.142]	(0.96 to 1.88)	(1.19 to 2.49)	(0.82 to 4.58)	[0.019]
Middle income	1.42	1.93	1.35	1.024	1.29	1.67	1.77	1.064
	(1.16 to 1.71)	(1.56 to 2.43)	(0.77 to 2.36)	[0.186]	(0.93 to 1.77)	(1.16 to 2.61)	(0.79 to 4.8)	[0.003]
RII	1.93	3.43	1.84	1.078	1.52	1.87	1.98	1.114
	(−2.92 to 6.78)	(−14.22 to 21.09)	(−1.99 to 5.67)	[0.096]	(−1.98 to 5.02)	(−9.48 to 13.21)	(−12.87 to 16.83)	[0.057]
*Absolute Inequalities*								
RD								
Low income	51.19	91.43	18.28	−2.648	10.93	17.12	11.26	0.791
	(29.89 to 73.1)	(72.5 to 110.46)	(−6.98 to 38.44)	[0.177]	(−1.59 to 23.89)	(6.28 to 29.13)	(−5.27 to 30.26)	[0.078]
Middle income	37.38	60.92	12.97	−2.130	9.27	17.50	13.36	0.649
	(16.31 to 56.58)	(42.38 to 82.78)	(−12.24 to 38.13)	[0.154]	(−2.63 to 20.05)	(4.9 to 31.58)	(−5.25 to 33.87)	[0.049]
SII	75.91	133.32	28.08	−3.685	16.18	16.70	16.70	1.189
	(−190.53 to 342.34)	(−285.28 to 551.93)	(−61.23 to 117.39)	[0.180]	(−69.24 to 101.59)	(−186.85 to 233.6)	(−150.98 to 184.37)	[0.091]

*Notes*: See File S1 for full results. U5MR, under-five mortality rate; NMR, neonatal mortality rate; CI, confidence interval; RR, rate ratio; RD, rate difference; RII, relative index of inequality; SII, slope index of inequality. 95% confidence intervals are reported in parentheses and *p*-values in brackets. The small number of observations and possible non-linear relationships implies that the trend estimates should be treated with caution. Additionally, since the bounds of the CI depend on the mean of mortality, comparisons over time must be treated cautiously.

As shown in Table 2, similar patterns are observed pertaining to the disparities in child mortality across rural-urban locations and regions. Absolute disparities in under-five mortality were found to have reduced over time while the opposite was estimated for neonatal mortality. However, most trends were not statistically significant. On the other hand, rising relative inequalities were statistically significant at conventional levels for both under-five and neonatal mortality. For example, RRs by rural-urban location for under-five children increased from 1.56 (95% CI 1.2 to 2) in 1989–90 to 2.41 (95% CI 1.1 to 3.6) in 2009–10, with an average biennial increase of 7.8%. Across the regions, this average increase ranged from 11.2% to 18.2% for under-five mortality and 16% to 17.9% for neonatal mortality.

**Table 2:** Inequalities in under-five and neonatal mortality (per 1,000 live births) by rural-urban and regions for selected years, with 95% confidence intervals and *p*-values for trend.

Equity Marker	U5MR				NMR			
	RR	95% CI	RD	95% CI	RR	95% CI	RD	95% CI
**Urban/Rural (base = Urban)**								
Rural								
1989–90	1.56	(1.18 to 2.04)	44.9	(18.68 to 66.73)	2.00	(1.31 to 3.28)	21.0	(9.62 to 31)
1999–2000	1.56	(1.23 to 1.86)	46.4	(22.8 to 62.34)	1.40	(1.01 to 2.04)	11.7	(0.22 to 22.02)
2009–10	2.41	(1.12 to 3.64)	31.1	(5.4 to 43.48)	2.91	(1.21 to 7.93)	19.1	(4.25 to 29.48)
Trend [*p*-value]	1.078	[0.012]	−0.562	[0.454]	1.066	[0.050]	0.109	[0.782]
**Island Division (base = Phnom Penh)**								
Plain								
1989–90	1.42	(0.9 to 2.24)	38.1	(−13.6 to 75)	1.61	(0.87 to 4.26)	17.8	(−6.73 to 38.64)
1999–2000	2.14	(1.22 to 3.52)	71.5	(23.1 to 102.4)	1.50	(0.64 to 4.62)	14.6	(−22.89 to 38.21)
2009–10	5.55	(0.61 to 10.04)	39.0	(−27.8 to 57.8)	6.10	(0.93 to 95.01)	29.4	(2.01 to 50.64)
Trend [*p*-value]	1.112	[0.001]	−1.187	[0.497]	1.160	[0.000]	0.673	[0.150]
Tonle Sap								
1989–90	1.25	(0.8 to 1.96)	23.0	(−28 to 59.3)	0.99	(0.54 to 2.62)	−0.4	(−23.02 to 18.65)
1999–2000	1.92	(1.1 to 3.1)	57.3	(10.9 to 83.1)	1.14	(0.52 to 3.45)	4.0	(−31.35 to 26.04)
2009–10	5.69	(0.65 to 9.58)	40.2	(−24.4 to 55.7)	4.33	(0.67 to 66.08)	19.2	(−5.32 to 36.45)
Trend [*p*-value]	1.113	[0.000]	−0.770	[0.655]	1.163	[0.004]	1.100	[0.180]
Coastal								
1989–90	0.97	(0.61 to 1.66)	−2.3	(−52.9 to 41)	1.20	(0.61 to 3.5)	6.0	(−17.92 to 30.24)
1999–2000	1.86	(1.06 to 3.2)	53.8	(6 to 90.5)	1.34	(0.56 to 4.41)	10.1	(−26.44 to 37.13)
2009–10	7.75	(0.86 to 17.1)	57.9	(−8 to 111.4)	3.75	(0.31 to 55.07)	15.9	(−10.39 to 45.99)
Trend [*p*-value]	1.182	[0.000]	4.103	[0.006]	1.179	[0.007]	1.464	[0.116]
Plateau/Mountain								
1989–90	1.45	(0.93 to 2.34)	41.3	(−10.4 to 84.1)	1.50	(0.8 to 4.01)	14.6	(−9.31 to 36.46)
1999–2000	2.06	(1.17 to 3.36)	66.3	(17.8 to 96)	1.42	(0.59 to 4.32)	12.2	(−25.33 to 35.3)
2009–10	7.21	(0.85 to 12.62)	53.2	(−8.8 to 81)	3.33	(0.44 to 40.02)	13.4	(−10.75 to 33.87)
Trend [*p*-value]	1.144	[0.000]	0.056	[0.977]	1.162	[0.006]	0.993	[0.189]

*Notes*: See File S1 for full results. U5MR, under-five mortality rate; NMR, neonatal mortality rate; RR, rate ratio; RD, rate difference; CI, confidence interval. The small number of observations and possible non-linear relationships implies that the trend estimates should be treated with caution.

Contrary to the general pattern, the estimates for two regions showed patterns of widening inequalities on both relative and absolute scales for under-five mortality. The upward trajectories, however, are only statistically significant for the trend in the Coastal region, with a *p*-value less than 0.01 associated with the positive trend. Full estimates of mortality, inequalities and trends are available in Tables S1–S4 in File S1.

## Discussion

Notwithstanding Cambodia's recent history of genocide under the Khmer Rouge (1975–1979) and internal conflict until the 1997 coup, this study confirms the noteworthy national reduction in under-five and neonatal mortality since 2000 [9]. These improvements are consistent with improvements in the maternal mortality ratio which decreased from 472 per 100,000 live births in 2000–2005 to 206 in 2006–2010 [9]. While it is not possible to make causal inferences from the available data, improvements are likely to be due to more than a decade of relative political and macroeconomic stability and high economic growth, increased female participation in the waged workforce and improved access to communications, transport infrastructure, education and potable water and sanitation [17], [32], [33]. These factors, particularly those related to the social determinants of health, are likely to have influenced health-related behaviours including changes in traditional birthing and feeding practices [4], [9], [12], [17], [19]. The government has also taken deliberate steps through its National Strategy for Reproductive and Sexual Health (2006–2010) [34] to address supply and demand side barriers in accessing maternal and child health services, which have also contributed to the observed reductions in under-five and neonatal mortality. Strategies have included demand-side financing policies, midwifery training and incentive schemes [17], [33], [35]. They have also been accompanied by strong malaria control and eradication strategies which have led to a decrease in the incidence of malaria including *Plasmodium falciparum*, which previously was one of the leading risk factors for under-five mortality [36].

Parallel to the observed national progress in U5MR, absolute inequalities by socioeconomic status show a decreasing, though not statistically significant trend, suggesting some gains in mortality across the wealth spectrum. This can be partly related to the observed reductions in disparities in access to family planning, safe abortion and immunization coverage among children across wealth groups [4], [9]. However, these gains have been tempered by stagnating chronic malnutrition, which has disproportionately affected the poor [9], [19], [37] and a widening equity gap for prevalence of under-five diarrhoea and coverage of postnatal care [4], [38].

Despite improvements in U5MR, the declines in the NMR seem to have stagnated since 2005 suggesting the need for increased investment in the more complex aspects of health system strengthening. Of concern are the persistent, and sometimes increasing, relative inequalities by socioeconomic status. The results show increasing trends in both relative and absolute inequality for neonates. These findings are consistent with the results from other studies [6], [12], [39] and reflect the general trend in Cambodia of increasing inequality across a number of socioeconomic markers [19] including a decreased concentration of landholding amongst the poor. We should note that increases in inequality for neonatal outcomes have been observed notwithstanding reductions in the equity gap for services like skilled birth attendant and antenatal care. This seems to suggest that similar to other settings, increases in coverage for disadvantaged populations might lack adequate levels of quality of care [40]. Therefore, more attention and resources should be devoted to measures targeting quality beyond extending coverage of critical interventions.

Across rural-urban locations similar patterns in child mortality inequality were observed as those seen by socioeconomic groups, which is not surprising since both equity markers show substantial overlap. For example, the distribution of households from the most recent DHS wave are such that over 90% of the households in the bottom two quintiles reside in rural areas, while approximately 75% of households in the top quintile dwell in urban areas. Interestingly, our results suggest that since Cambodia started experiencing increased economic growth (post-2000), relative urban/rural disparities have increased. This is also reflected in most other socioeconomic indicators [19]. This suggests that similar to many other low and middle income countries, economic growth has been accompanied by increased inequalities [41]. The observed increase in relative inequality across both wealth and rural/urban location of residence further points to the need for inclusive economic growth strategies as well as substantial political and financial investments in addressing important health system constraints in the provision of quality antennal and post-natal care, skilled birth attendants and intrapartum obstetric services to rural and other disadvantaged populations [4], [17], [19].

This study also revealed increasing relative and stable absolute inequalities amongst regions when compared with Phnom Penh. Given most of the urban population reside in Phnom Penh this is likely to be linked to rural/urban disparities discussed above. Households living in the regions, particularly in rural areas, may face persistent difficulties that relate to the social determinants of health and work against further under-five and neonatal mortality reduction.

An unexpected finding was the statistically significant sharp increase in absolute inequality for under-five mortality for coastal areas. The Coastal region has a thriving tourist industry and is neither the poorest nor the most remote of the regions. It is possible that this is due to systematic poverty and health disadvantage in some districts where local capacity is weak. The available data, however, did not allow us to explore this further with a reasonable level of robustness. The finding does warrant further quantitative and qualitative examination and suggests the need for disaggregated measures of health outcomes at the district level. Since the mountainous/plateau region has high poverty rates, undernutrition, low density of trained health workers and health services, future research should investigate whether several sources of potential biases exist in the DHS data. For example, it is possible that under-reporting occurs in the poorer and more remote mountainous/plateau region where 90% of births are estimated to take place at home. Under-reporting may be due to a number of issues including shame and stigma of reporting neonatal mortality especially if the birth was attended by a traditional birth attendant.

As with all similar mortality trend studies our analysis is subject to some limitations. First, a household asset index was used as a proxy measure for household wealth. This is a common measure in low-income settings where measures of income are often hard to obtain as many people work in informal labour markets with highly variable incomes often engaging in a mix of cash and non-monetary forms of trade. Similarly, accurate estimates of expenditure and consumption are often hard to obtain [42]. Nevertheless, there is some debate regarding the reliability and validity of the asset index as a proxy measure of household wealth [20], [21]. Second, as the DHS is a cross-sectional survey there may be disparity between reported household assets at the time of the survey, as opposed to the time of the reported child death [4]. Third, our analysis does not allow for examination of intra-region disparities. Finally, while pooling the data for various waves helps to reduce recall bias for overlapping periods and provides larger sub-national sample sizes, caution should be exercised when asserting the magnitude of inequalities and any small changes in mortality. Hence, we have emphasised the consistently observed trends in disparities, which are likely to be most valid.

To conclude, the trends revealed in this study suggest that across socioeconomic and geography markers under-five and neonatal mortality have improved in Cambodia although neo-natal mortality has stagnated. Geographical markers suggest entrenched and increased relative inequality in mortality. A number of factors have been discussed which may explain these geographical inequalities. The present study has helped highlight the importance of monitoring equity when evaluating child-health interventions. Cambodia's reliance on periodic household surveys to measure progress in under-five and neonatal mortality and health equity outcomes makes the quality of those surveys and subsequent analysis of particular importance. To meaningfully inform policy and planning decisions, data must be available at a disaggregated district level that complements the decentralised governance arrangements of the health system. This also requires improving survey quality to allow such analysis. Concurrently strengthening routine health information systems including birth and death registers is vital. Finally, concerted efforts are required to ensure that traditionally disadvantaged populations, start narrowing the inequality gap. This will be dependent on substantial health system investments to ensure the necessary services are accessible to all.

## Acknowledgments

The authors thank project staff at School of Population Health, The University of Queensland for their assistance in the development of this paper.

## Supporting Information

File S1 **Combined Supporting Information file containing:** **Box S1.** Measures of Inequality. **Table S1.** Inequalities in under-five and neonatal mortality (per 1,000 live births) by wealth for all years, with 95% confidence intervals and *p*-values for trend. **Table S2.** Inequalities in under-five and neonatal mortality (per 1,000 live births) by rural/urban location and regions for all years, with 95% confidence intervals and *p*-values for trend. **Table S3.** Under-five mortality rates per 1,000 live births. **Table S4.** Neonatal mortality rates per 1,000 live births.
(DOCX)
