# Supplementary material for: Entrenched Geographical and Socioeconomic Disparities in Child Mortality: Trends in Absolute and Relative Inequalities in Cambodia
Source: PLoS One. 2014 Oct 8;9(10):e109044. doi: 10.1371/journal.pone.0109044 (PMC4189958; doi:10.1371/journal.pone.0109044)
Supplement: File S1 — Combined Supporting Information file containing: Box S1. Measures of Inequality. Table S1. Inequalities in under-five and neonatal mortality (per 1,000 live births) by wealth for all years, with 95% confidence intervals and p-values for trend. Table S2. Inequalities in under-five and neonatal mortality (per 1,000 live births) by rural/urban location and regions for all years, with 95% confidence intervals and p-values for trend. Table S3. Under-five mortality rates per 1,000 live births. Table S4. Neonatal mortality rates per 1,000 live births. (DOCX) [file pone.0109044.s001.docx]

**FILE S1: SUPPORTING INFORMATION**

**Box S1:** Measures of Inequality

| *Rate Ratios (RR) and Rate Differences (RD)*  Rate ratios and differences compare the outcomes between two groups. Generally these two groups are in extreme situations (e.g. lowest and highest income groups); however, any bi-group comparisons can be made. These measures are computed by taking simple ratios of or the difference between the mortality rates of the two groups. Formally, let *j* = 1, 2 denote the two socioeconomic sub-populations, where the lowest socioeconomic group takes a value of 1 and the highest socioeconomic group a value of 2. The mortality rate for each group is denoted *MR_j_*. The rate ratio is computed as: $RR=\frac{MR_{1}}{MR_{2}}$   And the rate difference is calculated as: $RD=MR_{1}-MR_{2}$   *Relative Index of Inequality (RII) and Slope Index of Inequality (SII)*  The RII and SII are summary measures of inequality that take account of the population distribution categorised by socioeconomic status. Let, *j* = 1,…,*J* denote each ordered socioeconomic sub-population (e.g. by income). For each *j*, let *c_j_* be the fraction of the sample in group *j* or lower (with *c_0_* = 0 and *c_J_* = 1): that is, the cumulative relative position of the population ordered by the socioeconomic marker. For each *j*, let *ridit_j_* = (*c_j_* + *c_j-1_*)/2: that is, the midpoint of the relative rank. Again, denote the mortality rate for each *j* as *MR_j_*. The measures are computed by running a weighted least squares regression: $MR_{j}^{*}=\alpha+\beta ridit_{j}^{*}+\varepsilon_{j}$   where the variables enter the regression transformed by weights, which in our context are the total person-months in each socioeconomic category. Using this regression, the RII and SII are calculated as: $RII=\frac{\alpha}{\alpha+\beta}$   and $SII=-\beta$  |
| --- |

**Table S1:** Inequalities in under-five and neonatal mortality (per 1,000 live births) by wealth for all years, with 95% confidence intervals and *p*-values for trend

| **Equity Marker** |  | **Relative Inequalities** | | | |  | **Absolute Inequalities** | | | |
| --- | --- | --- | --- | --- | --- | --- | --- | --- | --- | --- |
|  |  | **RR** | **95% CI** | **RII** | **95% CI** |  | **RD** | **95% CI** | **SII** | **95% CI** |
| **Low Income** |  |  |  |  |  |  |  |  |  |  |
| *U5MR* |  |  |  |  |  |  |  |  |  |  |
| 1989-90 |  | 1.57 | (1.29; 1.91) | 1.93 | (-2.92; 6.78) |  | 51.2 | (29.9; 73.1) | 75.91 | (-190.53; 342.34) |
| 1991-92 |  | 1.88 | (1.55; 2.26) | 2.50 | (-6.56; 11.56) |  | 67.2 | (48.3; 84.7) | 99.28 | (-234.01; 432.56) |
| 1993-94 |  | 1.66 | (1.39; 1.94) | 2.16 | (1.95; 2.37) |  | 59.4 | (39.9; 76.2) | 88.94 | (78.92; 98.96) |
| 1995-96 |  | 1.76 | (1.47; 2.07) | 2.23 | (-4.58; 9.04) |  | 62.9 | (43.4; 80.4) | 91.88 | (-216.55; 400.31) |
| 1997-98 |  | 2.36 | (1.9; 2.86) | 3.17 | (-18.47; 24.81) |  | 85.9 | (67.3; 103.4) | 123.11 | (-442.66; 688.89) |
| 1999-2000 |  | 2.40 | (1.93; 2.93) | 3.43 | (-14.22; 21.09) |  | 91.4 | (72.5; 110.5) | 133.32 | (-285.28; 551.93) |
| 2001-02 |  | 2.13 | (1.57; 2.71) | 2.60 | (-18.99; 24.19) |  | 56.4 | (36.1; 74.8) | 79.25 | (-498.49; 656.99) |
| 2003-04 |  | 2.92 | (2.08; 4.01) | 4.81 | (-28.65; 38.27) |  | 70.3 | (51.4; 88) | 104.34 | (-192.9; 401.58) |
| 2005-06 |  | 2.01 | (1.28; 2.75) | 3.01 | (2.13; 3.89) |  | 38.6 | (15; 55.3) | 59.62 | (47.07; 72.17) |
| 2007-08 |  | 3.78 | (2.07; 6.03) | 11.61 | (-73.24; 96.45) |  | 54.8 | (33.5; 75.3) | 84.44 | (-13.06; 181.94) |
| 2009-10 |  | 1.50 | (0.87; 2.39) | 1.84 | (-1.99; 5.67) |  | 18.3 | (-7; 38.4) | 28.08 | (-61.23; 117.39) |
| Trend [*p*-value] |  | 1.037 | [0.142] | 1.078 | [0.096] |  | -2.648 | [0.177] | -3.685 | [0.180] |
|  |  |  |  |  |  |  |  |  |  |  |
| *NMR* |  |  |  |  |  |  |  |  |  |  |
| 1989-90 |  | 1.34 | (0.96; 1.88) | 1.52 | (-1.98; 5.02) |  | 10.9 | (-1.6; 23.9) | 16.18 | (-69.24; 101.59) |
| 1991-92 |  | 1.48 | (1.1; 2.08) | 1.71 | (-6.48; 9.91) |  | 12.8 | (3.1; 23) | 18.67 | (-138.06; 175.4) |
| 1993-94 |  | 1.12 | (0.86; 1.51) | 1.17 | (-0.89; 3.22) |  | 4.8 | (-6.3; 16.3) | 6.74 | (-70.3; 83.78) |
| 1995-96 |  | 1.27 | (0.96; 1.77) | 1.35 | (-4.8; 7.5) |  | 9.3 | (-1.7; 20.7) | 12.38 | (-171.06; 195.82) |
| 1997-98 |  | 2.07 | (1.46; 3.06) | 2.67 | (-11.86; 17.2) |  | 23.4 | (12.6; 34.1) | 33.51 | (-120.79; 187.81) |
| 1999-2000 |  | 1.65 | (1.19; 2.49) | 1.87 | (-9.48; 13.21) |  | 17.1 | (6.3; 29.1) | 23.37 | (-186.85; 233.6) |
| 2001-02 |  | 1.14 | (0.74; 1.79) | 1.16 | (-3.27; 5.59) |  | 4.4 | (-10.2; 18.1) | 5.17 | (-130.03; 140.36) |
| 2003-04 |  | 2.73 | (1.61; 5.71) | 2.88 | (-44.76; 50.52) |  | 23.1 | (12.2; 33.8) | 30.57 | (-355.79; 416.93) |
| 2005-06 |  | 1.50 | (0.87; 2.88) | 1.79 | (-1.99; 5.57) |  | 10.2 | (-4.1; 23.4) | 15.15 | (-36.29; 66.59) |
| 2007-08 |  | 4.68 | (2.16; 21.42) | 14.56 | (-365.53; 394.66) |  | 27.9 | (14.6; 44.3) | 42.39 | (-95.6; 180.38) |
| 2009-10 |  | 1.65 | (0.82; 4.58) | 1.98 | (-12.87; 16.83) |  | 11.3 | (-5.3; 30.3) | 16.70 | (-150.98; 184.37) |
| Trend [*p*-value] |  | 1.070 | [0.019] | 1.114 | [0.057] |  | 0.791 | [0.078] | 1.189 | [0.091] |
|  |  |  |  |  |  |  |  |  |  |  |
| **Middle Income** |  |  |  |  |  |  |  |  |  |  |
| *U5MR* |  |  |  |  |  |  |  |  |  |  |
| 1989-90 |  | 1.42 | (1.16; 1.71) | 1.93 | (-2.92; 6.78) |  | 37.4 | (16.3; 56.6) | 75.91 | (-190.53; 342.34) |
| 1991-92 |  | 1.63 | (1.33; 1.94) | 2.50 | (-6.56; 11.56) |  | 47.9 | (29.1; 65.1) | 99.28 | (-234.01; 432.56) |
| 1993-94 |  | 1.32 | (1.11; 1.58) | 2.16 | (1.95; 2.37) |  | 28.9 | (10.7; 46.7) | 88.94 | (78.92; 98.96) |
| 1995-96 |  | 1.53 | (1.25; 1.8) | 2.23 | (-4.58; 9.04) |  | 43.6 | (22.9; 59.7) | 91.88 | (-216.55; 400.31) |
| 1997-98 |  | 2.03 | (1.61; 2.5) | 3.17 | (-18.47; 24.81) |  | 65.4 | (45.1; 83.8) | 123.11 | (-442.66; 688.89) |
| 1999-2000 |  | 1.93 | (1.56; 2.43) | 3.43 | (-14.22; 21.09) |  | 60.9 | (42.4; 82.8) | 133.32 | (-285.28; 551.93) |
| 2001-02 |  | 2.05 | (1.48; 2.72) | 2.60 | (-18.99; 24.19) |  | 52.6 | (30.6; 73.5) | 79.25 | (-498.49; 656.99) |
| 2003-04 |  | 2.25 | (1.54; 3.18) | 4.81 | (-28.65; 38.27) |  | 45.5 | (26.1; 64.5) | 104.34 | (-192.9; 401.58) |
| 2005-06 |  | 1.47 | (0.93; 2.1) | 3.01 | (2.13; 3.89) |  | 18.0 | (-4; 34.6) | 59.62 | (47.07; 72.17) |
| 2007-08 |  | 2.54 | (1.33; 4.16) | 11.61 | (-73.24; 96.45) |  | 30.3 | (10.2; 48.3) | 84.44 | (-13.06; 181.94) |
| 2009-10 |  | 1.35 | (0.77; 2.36) | 1.84 | (-1.99; 5.67) |  | 13.0 | (-12.2; 38.1) | 28.08 | (-61.23; 117.39) |
| Trend [*p*-value] |  | 1.024 | [0.186] | 1.078 | [0.096] |  | -2.130 | [0.154] | -3.685 | [0.180] |
|  |  |  |  |  |  |  |  |  |  |  |
| *NMR* |  |  |  |  |  |  |  |  |  |  |
| 1989-90 |  | 1.29 | (0.93; 1.77) | 1.52 | (-1.98; 5.02) |  | 9.3 | (-2.6; 20) | 16.18 | (-69.24; 101.59) |
| 1991-92 |  | 1.50 | (1.08; 2.1) | 1.71 | (-6.48; 9.91) |  | 13.4 | (2.5; 24.4) | 18.67 | (-138.06; 175.4) |
| 1993-94 |  | 1.14 | (0.86; 1.54) | 1.17 | (-0.89; 3.22) |  | 5.8 | (-6.6; 18.5) | 6.74 | (-70.3; 83.78) |
| 1995-96 |  | 1.38 | (0.98; 1.92) | 1.35 | (-4.8; 7.5) |  | 12.7 | (-0.7; 24.6) | 12.38 | (-171.06; 195.82) |
| 1997-98 |  | 1.82 | (1.23; 2.64) | 2.67 | (-11.86; 17.2) |  | 17.8 | (6.5; 28.2) | 33.51 | (-120.79; 187.81) |
| 1999-2000 |  | 1.67 | (1.16; 2.61) | 1.87 | (-9.48; 13.21) |  | 17.5 | (4.9; 31.6) | 23.37 | (-186.85; 233.6) |
| 2001-02 |  | 1.27 | (0.8; 2.06) | 1.16 | (-3.27; 5.59) |  | 8.3 | (-7.8; 23.7) | 5.17 | (-130.03; 140.36) |
| 2003-04 |  | 3.14 | (1.73; 6.81) | 2.88 | (-44.76; 50.52) |  | 28.6 | (14.4; 44.5) | 30.57 | (-355.79; 416.93) |
| 2005-06 |  | 1.34 | (0.76; 2.5) | 1.79 | (-1.99; 5.57) |  | 7.0 | (-6.6; 20.5) | 15.15 | (-36.29; 66.59) |
| 2007-08 |  | 3.58 | (1.55; 16.55) | 14.56 | (-365.53; 394.66) |  | 19.6 | (6.4; 34.7) | 42.39 | (-95.6; 180.38) |
| 2009-10 |  | 1.77 | (0.79; 4.8) | 1.98 | (-12.87; 16.83) |  | 13.4 | (-5.3; 33.9) | 16.70 | (-150.98; 184.37) |
| Trend [*p*-value] |  | 1.064 | [0.003] | 1.114 | [0.057] |  | 0.649 | [0.049] | 1.189 | [0.091] |

*Notes*: U5MR, under-five mortality rate; NMR, neonatal mortality rate; CI, confidence interval; RR, rate ratio; RD, rate difference; RII, relative index of inequality; SII, slope index of inequality. The small number of observations and possible non-linear relationships implies that the trend estimates should be treated with caution. The RIIs and SIIs are the same for both low and middle income as they are computed jointly.

**Table S2:** Inequalities in under-five and neonatal mortality (per 1,000 live births) by rural/urban location and regions for all years, with 95% confidence intervals and *p*-values for trend

| **Equity Marker** |  | **U5MR** | | | |  | **NMR** | | | |
| --- | --- | --- | --- | --- | --- | --- | --- | --- | --- | --- |
|  |  | **RR** | **95% CI** | **RD** | **95% CI** |  | **RR** | **95% CI** | **RD** | **95% CI** |
| **Urban/Rural (base = Urban)** | | | |  |  |  |  |  |  |  |
| Rural |  |  |  |  |  |  |  |  |  |  |
| 1989-90 |  | 1.56 | (1.18; 2.04) | 44.9 | (18.68; 66.73) |  | 2.00 | (1.31; 3.28) | 21.0 | (9.62; 31) |
| 1991-92 |  | 1.43 | (1.14; 1.77) | 36.5 | (14.12; 54.1) |  | 1.66 | (1.16; 2.43) | 15.0 | (5.23; 23.12) |
| 1993-94 |  | 1.43 | (1.19; 1.72) | 38.0 | (19.75; 54.44) |  | 1.87 | (1.39; 2.63) | 21.9 | (12.46; 30.25) |
| 1995-96 |  | 1.72 | (1.37; 2.09) | 53.7 | (33.52; 69.15) |  | 1.47 | (1.03; 2.14) | 13.8 | (1.22; 24.56) |
| 1997-98 |  | 1.43 | (1.18; 1.73) | 37.7 | (18.33; 54.86) |  | 1.43 | (1; 2.17) | 11.7 | (0.06; 22.18) |
| 1999-2000 |  | 1.56 | (1.23; 1.86) | 46.4 | (22.8; 62.34) |  | 1.40 | (1.01; 2.04) | 11.7 | (0.22; 22.02) |
| 2001-02 |  | 1.30 | (0.98; 1.66) | 21.7 | (-2.1; 39.4) |  | 1.23 | (0.78; 1.97) | 6.9 | (-8.86; 19.48) |
| 2003-04 |  | 2.18 | (1.53; 2.77) | 47.6 | (29.15; 60.12) |  | 3.29 | (1.89; 6.7) | 25.0 | (15.4; 34.02) |
| 2005-06 |  | 4.54 | (2.13; 7.07) | 52.9 | (33.61; 62.65) |  | 4.63 | (2.2; 19.03) | 23.9 | (13.95; 32.83) |
| 2007-08 |  | 2.48 | (1.28; 3.31) | 34.1 | (11.71; 45.21) |  | 2.13 | (1; 4.93) | 14.5 | (0.09; 25.53) |
| 2009-10 |  | 2.41 | (1.12; 3.64) | 31.1 | (5.4; 43.48) |  | 2.91 | (1.21; 7.93) | 19.1 | (4.25; 29.48) |
| Trend [*p*-value] |  | 1.078 | [0.012] | -0.562 | [0.454] |  | 1.066 | [0.050] | 0.109 | [0.782] |
|  |  |  |  |  |  |  |  |  |  |  |
| **Island Division (base = Phnom Penh)** | | | |  |  |  |  |  |  |  |
| Plain |  |  |  |  |  |  |  |  |  |  |
| 1989-90 |  | 1.42 | (0.9; 2.24) | 38.1 | (-13.6; 75.05) |  | 1.61 | (0.87; 4.26) | 17.8 | (-6.73; 38.64) |
| 1991-92 |  | 1.69 | (1.09; 2.78) | 46.6 | (8.85; 74.31) |  | 1.67 | (0.89; 4.08) | 15.0 | (-4.13; 30.49) |
| 1993-94 |  | 1.67 | (1.11; 2.42) | 50.7 | (11.99; 78.16) |  | 2.89 | (1.7; 6.77) | 33.5 | (19.7; 47.17) |
| 1995-96 |  | 2.04 | (1.31; 3.36) | 65.6 | (30.28; 95.59) |  | 1.42 | (0.74; 4.25) | 13.1 | (-14.2; 35.12) |
| 1997-98 |  | 3.30 | (1.78; 4.78) | 88.2 | (53.38; 107.06) |  | 2.74 | (1.23; 8.19) | 25.2 | (7.34; 37.61) |
| 1999-2000 |  | 2.14 | (1.22; 3.52) | 71.5 | (23.09; 102.39) |  | 1.50 | (0.64; 4.62) | 14.6 | (-22.89; 38.21) |
| 2001-02 |  | 2.10 | (1.08; 3.24) | 50.4 | (6.61; 74.59) |  | 1.30 | (0.67; 3.12) | 8.9 | (-17.23; 29.58) |
| 2003-04 |  | 3.69 | (1.3; 5.42) | 60.7 | (17.67; 75.79) |  | 7.39 | (1.84; 21.34) | 29.7 | (13.39; 42.23) |
| 2005-06 |  | 2.03 | (0.86; 15.77) | 27.6 | (-9.36; 60.13) |  | 6.40 | (1.03; 96.52) | 20.6 | (3.77; 35.03) |
| 2007-08 |  | 4.04 | (0.81; 10.85) | 42.0 | (-11.98; 62.46) |  | 5.42 | (1.31; 7.04) | 24.5 | (7.83; 41.81) |
| 2009-10 |  | 5.55 | (0.61; 10.04) | 39.0 | (-27.8; 57.83) |  | 6.10 | (0.93; 95.01) | 29.4 | (2.01; 50.64) |
| Trend [*p*-value] |  | 1.112 | [0.001] | -1.187 | [0.497] |  | 1.160 | [0.000] | 0.673 | [0.150] |
|  |  |  |  |  |  |  |  |  |  |  |
| Tonle Sap |  |  |  |  |  |  |  |  |  |  |
| 1989-90 |  | 1.25 | (0.8; 1.96) | 23.0 | (-28.03; 59.35) |  | 0.99 | (0.54; 2.62) | -0.4 | (-23.02; 18.65) |
| 1991-92 |  | 1.87 | (1.2; 3.05) | 59.0 | (20.32; 87.94) |  | 1.66 | (0.91; 3.65) | 14.7 | (-3.56; 28.05) |
| 1993-94 |  | 1.59 | (1.08; 2.25) | 45.0 | (9.28; 69.6) |  | 2.33 | (1.36; 5.26) | 23.6 | (10.1; 35.64) |
| 1995-96 |  | 1.95 | (1.29; 3.22) | 60.0 | (27.06; 87.73) |  | 1.37 | (0.73; 4.11) | 11.6 | (-14.59; 33.11) |
| 1997-98 |  | 3.05 | (1.65; 4.56) | 78.6 | (44.89; 95.26) |  | 2.52 | (1.18; 7.25) | 22.0 | (5.28; 33.66) |
| 1999-2000 |  | 1.92 | (1.1; 3.1) | 57.3 | (10.86; 83.12) |  | 1.14 | (0.52; 3.45) | 4.0 | (-31.35; 26.04) |
| 2001-02 |  | 1.99 | (1.05; 3.1) | 45.4 | (3.64; 65.46) |  | 1.09 | (0.59; 2.85) | 2.8 | (-20.08; 23.61) |
| 2003-04 |  | 3.60 | (1.28; 5.44) | 58.5 | (17.4; 71.5) |  | 6.61 | (1.77; 18.36) | 26.1 | (11.86; 36.63) |
| 2005-06 |  | 2.46 | (1; 19.63) | 39.0 | (0.22; 71.66) |  | 8.77 | (1.47; 113.81) | 29.6 | (11.25; 42.73) |
| 2007-08 |  | 3.17 | (0.63; 8.08) | 29.9 | (-25.08; 44.27) |  | 3.44 | (0.74; 4.36) | 13.5 | (-2.56; 26.25) |
| 2009-10 |  | 5.69 | (0.65; 9.58) | 40.2 | (-24.43; 55.68) |  | 4.33 | (0.67; 66.08) | 19.2 | (-5.32; 36.45) |
| Trend [*p*-value] |  | 1.113 | [0.000] | -0.770 | [0.655] |  | 1.163 | [0.004] | 1.100 | [0.180] |
|  |  |  |  |  |  |  |  |  |  |  |
| Coastal |  |  |  |  |  |  |  |  |  |  |
| 1989-90 |  | 0.97 | (0.61; 1.66) | -2.3 | (-52.9; 41) |  | 1.20 | (0.61; 3.5) | 6.0 | (-17.92; 30.24) |
| 1991-92 |  | 1.61 | (1; 2.8) | 40.9 | (-0.3; 76.7) |  | 2.03 | (1.02; 4.74) | 22.9 | (0.75; 40.97) |
| 1993-94 |  | 1.32 | (0.87; 2) | 24.4 | (-13.1; 57) |  | 2.10 | (1.18; 5) | 19.6 | (4.62; 37.1) |
| 1995-96 |  | 1.39 | (0.9; 2.49) | 24.8 | (-8.8; 61.9) |  | 0.95 | (0.46; 2.84) | -1.7 | (-27.42; 20.68) |
| 1997-98 |  | 3.03 | (1.57; 4.74) | 77.7 | (37.3; 106.4) |  | 2.74 | (1.09; 8.76) | 25.2 | (2.46; 44.17) |
| 1999-2000 |  | 1.86 | (1.06; 3.2) | 53.8 | (6; 90.5) |  | 1.34 | (0.56; 4.41) | 10.1 | (-26.44; 37.13) |
| 2001-02 |  | 1.69 | (0.85; 2.92) | 31.6 | (-11.6; 67.7) |  | 1.33 | (0.6; 3.65) | 9.7 | (-18.14; 39.22) |
| 2003-04 |  | 2.61 | (0.9; 4.51) | 36.2 | (-5.9; 60.8) |  | 7.34 | (1.65; 23.5) | 29.5 | (9.24; 48.51) |
| 2005-06 |  | 3.29 | (1.33; 25.28) | 61.0 | (18.4; 108.2) |  | 9.82 | (1.46; 139.74) | 33.6 | (9.53; 57.69) |
| 2007-08 |  | 5.11 | (0.99; 14.99) | 56.8 | (0; 108.2) |  | 6.15 | (0.64; 12.67) | 28.5 | (-2.2; 71.58) |
| 2009-10 |  | 7.75 | (0.86; 17.1) | 57.9 | (-8; 111.4) |  | 3.75 | (0.31; 55.07) | 15.9 | (-10.39; 45.99) |
| Trend [*p*-value] |  | 1.182 | [0.000] | 4.103 | [0.006] |  | 1.179 | [0.007] | 1.464 | [0.116] |
|  |  |  |  |  |  |  |  |  |  |  |
| Plateau/Mountain |  |  |  |  |  |  |  |  |  |  |
| 1989-90 |  | 1.45 | (0.93; 2.34) | 41.3 | (-10.4; 84.1) |  | 1.50 | (0.8; 4.01) | 14.6 | (-9.31; 36.46) |
| 1991-92 |  | 1.93 | (1.23; 3.21) | 62.9 | (22.9; 96.3) |  | 1.28 | (0.65; 3.12) | 6.2 | (-13.5; 22.19) |
| 1993-94 |  | 1.86 | (1.27; 2.62) | 65.5 | (29.4; 94.1) |  | 2.56 | (1.47; 5.92) | 27.6 | (13.15; 42.46) |
| 1995-96 |  | 2.22 | (1.47; 3.65) | 76.9 | (43.3; 109.3) |  | 1.32 | (0.69; 3.7) | 10.0 | (-17.45; 32.9) |
| 1997-98 |  | 3.82 | (2.04; 5.65) | 108.1 | (70.3; 132) |  | 2.88 | (1.27; 8.95) | 27.2 | (7.82; 42.72) |
| 1999-2000 |  | 2.06 | (1.17; 3.36) | 66.3 | (17.8; 96) |  | 1.42 | (0.59; 4.32) | 12.2 | (-25.33; 35.3) |
| 2001-02 |  | 2.19 | (1.1; 3.42) | 54.5 | (8.4; 79.9) |  | 1.23 | (0.62; 3.18) | 6.7 | (-19.04; 29.54) |
| 2003-04 |  | 5.31 | (1.86; 7.78) | 97.1 | (53.2; 116.7) |  | 9.41 | (2.3; 26.42) | 39.1 | (22.18; 53.94) |
| 2005-06 |  | 3.20 | (1.3; 26.66) | 58.6 | (19.1; 96.8) |  | 7.85 | (1.26; 88.34) | 26.1 | (8.08; 42.28) |
| 2007-08 |  | 5.20 | (1.04; 13.32) | 58.1 | (2.5; 86.5) |  | 5.81 | (1.32; 7.82) | 26.7 | (8.01; 45.96) |
| 2009-10 |  | 7.21 | (0.85; 12.62) | 53.2 | (-8.8; 81) |  | 3.33 | (0.44; 40.02) | 13.4 | (-10.75; 33.87) |
| Trend [*p*-value] |  | 1.144 | [0.000] | 0.056 | [0.977] |  | 1.162 | [0.006] | 0.993 | [0.189] |

*Notes*: U5MR, under-five mortality rate; NMR, neonatal mortality rate; CI, confidence interval; RR, rate ratio; RD, rate difference. The small number of observations and possible non-linear relationships implies that the trend estimates should be treated with caution. The RIIs and SIIs are the same for both low and middle income as they are computed jointly.

**Table S3:** Under-five mortality rates per 1,000 live births

| **Equity Marker** | **U5MR** | **95% CI** | **U5MR** | **95% CI** | **U5MR** | **95% CI** |
| --- | --- | --- | --- | --- | --- | --- |
| **National** |  |  |  |  |  |  |
| 1989-90 | 119.6 | (111.4; 128.4) |  |  |  |  |
| 1991-92 | 116.1 | (108.7; 124.3) |  |  |  |  |
| 1993-94 | 121.5 | (114.1; 129.1) |  |  |  |  |
| 1995-96 | 121.2 | (113.9; 129.7) |  |  |  |  |
| 1997-98 | 119.9 | (112.1; 127.9) |  |  |  |  |
| 1999-2000 | 122.9 | (114.5; 132.7) |  |  |  |  |
| 2001-02 | 90.1 | (82.2; 99.7) |  |  |  |  |
| 2003-04 | 81.1 | (73.1; 90.6) |  |  |  |  |
| 2005-06 | 60.2 | (53; 69.3) |  |  |  |  |
| 2007-08 | 51.8 | (44.2; 62.3) |  |  |  |  |
| 2009-10 | 48.1 | (40.2; 59.9) |  |  |  |  |
| Trend [*p*-value] | -8.26 | [0.000] |  |  |  |  |
|  |  |  |  |  |  |  |
| **Urban/Rural** | Urban |  | Rural |  |  |  |
| 1989-90 | 80.7 | (62.8; 105.8) | 125.6 | (117.1; 136.1) |  |  |
| 1991-92 | 84.5 | (69.6; 105.1) | 121.0 | (112.8; 130.3) |  |  |
| 1993-94 | 88.6 | (74.6; 105.5) | 126.5 | (119; 134.9) |  |  |
| 1995-96 | 74.7 | (62.5; 92.6) | 128.4 | (119.9; 137.3) |  |  |
| 1997-98 | 87.1 | (71.6; 104.1) | 124.8 | (116.2; 134.9) |  |  |
| 1999-2000 | 82.6 | (70.4; 103.5) | 129.1 | (119.6; 140.8) |  |  |
| 2001-02 | 71.4 | (56.5; 93.6) | 93.1 | (83.2; 103.4) |  |  |
| 2003-04 | 40.4 | (32.4; 57.5) | 87.9 | (78.8; 98.7) |  |  |
| 2005-06 | 15.0 | (9.1; 31.1) | 67.9 | (58.7; 78.7) |  |  |
| 2007-08 | 23.1 | (18.9; 43.9) | 57.2 | (49; 69.8) |  |  |
| 2009-10 | 22.0 | (15; 45.5) | 53.1 | (42.2; 66.1) |  |  |
| Trend [*p*-value] | -7.68 | [0.000] | -8.24 | [0.000] |  |  |
|  |  |  |  |  |  |  |
| **Wealth** | Low Income |  | Middle Income | | High Income | |
| 1989-90 | 140.9 | (127; 158.4) | 127.0 | (113.8; 142.9) | 89.7 | (78.1; 106.7) |
| 1991-92 | 143.5 | (131.8; 158.4) | 124.2 | (113; 139.1) | 76.3 | (65.1; 91.1) |
| 1993-94 | 149.3 | (137.7; 162.6) | 118.8 | (107.2; 133.3) | 89.9 | (78.4; 103.6) |
| 1995-96 | 145.5 | (132.3; 159.6) | 126.3 | (112.6; 139.1) | 82.6 | (71.8; 97.6) |
| 1997-98 | 149.1 | (135.1; 164.4) | 128.6 | (115.6; 144.6) | 63.2 | (53.4; 77.3) |
| 1999-2000 | 156.8 | (142.2; 172.4) | 126.3 | (112.4; 145.2) | 65.4 | (55.1; 79.4) |
| 2001-02 | 106.4 | (94.7; 123.4) | 102.6 | (87.1; 122.2) | 50.0 | (40.6; 67) |
| 2003-04 | 106.9 | (93.3; 122.1) | 82.1 | (68.5; 100.8) | 36.6 | (27.6; 50.7) |
| 2005-06 | 76.7 | (65.2; 91.7) | 56.1 | (44.8; 72) | 38.1 | (29.5; 58.1) |
| 2007-08 | 74.5 | (61.2; 95.3) | 50.0 | (37.2; 68) | 19.7 | (12.8; 33.9) |
| 2009-10 | 55.1 | (41.1; 73.1) | 49.8 | (36.1; 73.3) | 36.8 | (25.6; 58.6) |
| Trend [*p*-value] | -9.48 | [0.001] | -8.96 | [0.000] | -6.83 | [0.000] |
|  |  |  |  |  |  |  |
| **Island** | Plain |  | Tonle Sap |  | Coastal |  |
| 1989-90 | 129.7 | (116.3; 146.5) | 114.7 | (104.2; 129.3) | 89.4 | (71.5; 116.4) |
| 1991-92 | 114.1 | (101.4; 129.1) | 126.5 | (115.1; 140.3) | 108.4 | (91.5; 137.2) |
| 1993-94 | 126.9 | (114.6; 142.2) | 121.2 | (111.4; 133.2) | 100.6 | (85.5; 124.5) |
| 1995-96 | 128.9 | (115.4; 143.4) | 123.3 | (111.6; 136.6) | 88.1 | (70.8; 113.2) |
| 1997-98 | 126.5 | (112.6; 143.7) | 116.9 | (106.2; 129.8) | 116.0 | (95.4; 143.1) |
| 1999-2000 | 134.0 | (117.2; 154.2) | 119.8 | (107.2; 134.4) | 116.3 | (97.1; 147.2) |
| 2001-02 | 96.3 | (81.5; 118.5) | 91.2 | (81.1; 107.7) | 77.5 | (60; 115.4) |
| 2003-04 | 83.2 | (69.1; 103.3) | 81.0 | (71; 95.9) | 58.7 | (44.7; 90) |
| 2005-06 | 54.2 | (43.3; 72) | 65.6 | (53.8; 82) | 87.6 | (61.7; 126.9) |
| 2007-08 | 55.8 | (43.1; 78.8) | 43.7 | (33.9; 59.5) | 70.6 | (47.2; 130.7) |
| 2009-10 | 47.6 | (33.9; 73.1) | 48.8 | (39; 69.3) | 66.5 | (43.6; 132.8) |
| Trend [*p*-value] | -8.94 | [0.001] | -8.52 | [0.000] | -3.65 | [0.005] |
|  |  |  |  |  |  |  |
|  | Plateau/Mountain | | Phnom Penh |  |  |  |
| 1989-90 | 133.0 | (114; 157.8) | 91.7 | (58.4; 143.3) |  |  |
| 1991-92 | 130.5 | (114.2; 154.8) | 67.5 | (41.9; 105.3) |  |  |
| 1993-94 | 141.7 | (125; 162.8) | 76.2 | (55.5; 111.8) |  |  |
| 1995-96 | 140.2 | (123.4; 161.5) | 63.3 | (38.6; 94.3) |  |  |
| 1997-98 | 146.4 | (128.6; 169.8) | 38.3 | (25.9; 71.7) |  |  |
| 1999-2000 | 128.8 | (111.3; 150.3) | 62.5 | (38.8; 108.1) |  |  |
| 2001-02 | 100.3 | (82.5; 122.7) | 45.9 | (30.9; 89.4) |  |  |
| 2003-04 | 119.6 | (100.9; 144.2) | 22.5 | (15.6; 64.7) |  |  |
| 2005-06 | 85.3 | (67.9; 110) | 26.6 | (0.3; 63.1) |  |  |
| 2007-08 | 71.9 | (55.7; 103.5) | 13.8 | (6; 69.9) |  |  |
| 2009-10 | 61.8 | (45.5; 97.8) | 8.6 | (0.7; 75.7) |  |  |
| Trend [*p*-value] | -7.70 | [0.002] | -7.75 | [0.000] |  |  |

*Notes*: U5MR, under-five mortality rate; CI, confidence interval; Quin., Quintile. The small number of observations and possible non-linear relationships implies that the trend estimates should be treated with caution.

**Table S4:** Neonatal mortality rates per 1,000 live births

| **Equity Marker** | **NMR** | **95% CI** | **NMR** | **95% CI** | **NMR** | **95% CI** |
| --- | --- | --- | --- | --- | --- | --- |
| **National** |  |  |  |  |  |  |
| 1989-90 | 39.2 | (34.3; 44.1) |  |  |  |  |
| 1991-92 | 35.7 | (31.4; 40.1) |  |  |  |  |
| 1993-94 | 44.1 | (39.4; 49.1) |  |  |  |  |
| 1995-96 | 41.6 | (37.4; 46.7) |  |  |  |  |
| 1997-98 | 37.4 | (32.7; 41.9) |  |  |  |  |
| 1999-2000 | 39.0 | (34.3; 44.3) |  |  |  |  |
| 2001-02 | 35.6 | (29.3; 42) |  |  |  |  |
| 2003-04 | 32.2 | (26.4; 38.1) |  |  |  |  |
| 2005-06 | 26.9 | (21.4; 33.5) |  |  |  |  |
| 2007-08 | 25.2 | (19.3; 31.8) |  |  |  |  |
| 2009-10 | 26.0 | (19; 34.9) |  |  |  |  |
| Trend [*p*-value] | -1.64 | [0.003] |  |  |  |  |
|  |  |  |  |  |  |  |
| **Urban/Rural** | Urban |  | Rural |  |  |  |
| 1989-90 | 21.0 | (12.6; 31.4) | 42.0 | (36.6; 47.9) |  |  |
| 1991-92 | 22.7 | (15.9; 31.1) | 37.7 | (33.1; 43.1) |  |  |
| 1993-94 | 25.1 | (17.9; 33) | 47.0 | (41.8; 52) |  |  |
| 1995-96 | 29.7 | (20.6; 40.1) | 43.5 | (38.1; 49.1) |  |  |
| 1997-98 | 27.2 | (18.3; 38) | 39.0 | (34; 44.5) |  |  |
| 1999-2000 | 28.9 | (19.8; 38.7) | 40.6 | (35.3; 46.3) |  |  |
| 2001-02 | 29.7 | (18.7; 43.3) | 36.6 | (29.3; 43) |  |  |
| 2003-04 | 10.9 | (4.9; 17.8) | 35.9 | (29.6; 43) |  |  |
| 2005-06 | 6.6 | (1.8; 13.3) | 30.5 | (23.9; 38) |  |  |
| 2007-08 | 12.9 | (5.6; 24.9) | 27.4 | (20.2; 36.4) |  |  |
| 2009-10 | 10.0 | (3.9; 22) | 29.1 | (20.6; 38.7) |  |  |
| Trend [*p*-value] | -1.68 | [0.029] | -1.57 | [0.003] |  |  |
|  |  |  |  |  |  |  |
| **Wealth** | Low Income |  | Middle Income | | High Income | |
| 1989-90 | 43.2 | (34.8; 52.9) | 41.5 | (34; 49.5) | 32.2 | (25; 41) |
| 1991-92 | 39.4 | (32.9; 47.2) | 39.9 | (31.6; 48.7) | 26.6 | (19.8; 33.5) |
| 1993-94 | 45.2 | (38.6; 52.4) | 46.2 | (37.9; 54.9) | 40.4 | (32.1; 49.1) |
| 1995-96 | 43.0 | (36.6; 51.9) | 46.4 | (38.4; 55.4) | 33.7 | (25.4; 43.2) |
| 1997-98 | 45.2 | (37.9; 53.3) | 39.7 | (31.9; 47.6) | 21.8 | (15.4; 29.4) |
| 1999-2000 | 43.3 | (36.4; 51.9) | 43.7 | (34.5; 55) | 26.2 | (18.4; 34.8) |
| 2001-02 | 35.6 | (27.4; 45.1) | 39.5 | (28.4; 51.8) | 31.2 | (20.9; 43.1) |
| 2003-04 | 36.5 | (29.2; 45.3) | 41.9 | (30.3; 55.7) | 13.3 | (6.8; 21.4) |
| 2005-06 | 30.6 | (22; 41.1) | 27.5 | (18.2; 37.9) | 20.5 | (12.1; 31.8) |
| 2007-08 | 35.5 | (23.7; 50.6) | 27.2 | (16.6; 41.3) | 7.6 | (1.5; 14.5) |
| 2009-10 | 28.6 | (18.1; 42.9) | 30.7 | (15.9; 50.4) | 17.3 | (7.4; 29.4) |
| Trend [*p*-value] | -1.41 | [0.002] | -1.55 | [0.015] | -2.20 | [0.002] |
|  |  |  |  |  |  |  |
|  |  |  |  |  |  |  |
|  |  |  |  |  |  |  |
|  |  |  |  |  |  |  |
|  |  |  |  |  |  |  |
|  |  |  |  |  |  |  |
| **Island** | Plain |  | Tonle Sap |  | Coastal |  |
| 1989-90 | 47.3 | (38.9; 57.3) | 29.1 | (23.3; 36) | 35.4 | (23.9; 51.7) |
| 1991-92 | 37.3 | (29.5; 45.8) | 36.9 | (30.6; 44.1) | 45.2 | (30.5; 60.7) |
| 1993-94 | 51.2 | (43; 61) | 41.3 | (34.9; 47.7) | 37.3 | (26.5; 51.2) |
| 1995-96 | 44.4 | (36.2; 52.9) | 42.9 | (35.1; 50.6) | 29.6 | (18.8; 40.9) |
| 1997-98 | 39.7 | (32; 48.9) | 36.5 | (29.9; 43.7) | 39.7 | (25.7; 55.7) |
| 1999-2000 | 44.0 | (35.5; 54.5) | 33.4 | (26.5; 41.3) | 39.5 | (26.2; 55.3) |
| 2001-02 | 38.4 | (27.5; 50.6) | 32.3 | (25; 41.8) | 39.2 | (22.8; 64.8) |
| 2003-04 | 34.3 | (23.3; 47.5) | 30.7 | (23.4; 41.1) | 34.1 | (20.7; 53.1) |
| 2005-06 | 24.4 | (14.4; 36.4) | 33.4 | (23; 45.5) | 37.4 | (17.4; 59.4) |
| 2007-08 | 30.0 | (18.7; 44.5) | 19.0 | (11.1; 27.7) | 34.1 | (13.2; 77.7) |
| 2009-10 | 35.1 | (19.3; 52.7) | 25.0 | (16; 37.9) | 21.6 | (7.2; 46) |
| Trend [*p*-value] | -1.74 | [0.007] | -1.31 | [0.087] | -0.95 | [0.118] |
|  |  |  |  |  |  |  |
|  | Plateau/Mountain | | Phnom Penh |  |  |  |
| 1989-90 | 44.1 | (32.2; 56.1) | 29.5 | (11.7; 51.4) |  |  |
| 1991-92 | 28.5 | (19.9; 39.1) | 22.3 | (8.9; 39.4) |  |  |
| 1993-94 | 45.3 | (35; 57.1) | 17.7 | (8.1; 28.3) |  |  |
| 1995-96 | 41.4 | (31; 52.7) | 31.4 | (9.8; 55.7) |  |  |
| 1997-98 | 41.7 | (31.4; 54.5) | 14.5 | (5.1; 29.8) |  |  |
| 1999-2000 | 41.6 | (30.8; 52.4) | 29.4 | (10.9; 67.8) |  |  |
| 2001-02 | 36.3 | (24.2; 50.1) | 29.6 | (10.8; 52.3) |  |  |
| 2003-04 | 43.8 | (32; 59.5) | 4.6 | (2.1; 16.1) |  |  |
| 2005-06 | 29.9 | (17.4; 43.8) | 3.8 | (0; 18.5) |  |  |
| 2007-08 | 32.2 | (19.9; 50.1) | 5.5 | (0; 18.4) |  |  |
| 2009-10 | 19.2 | (8.4; 36.4) | 5.8 | (0; 28.6) |  |  |
| Trend [*p*-value] | -1.42 | [0.085] | -2.41 | [0.001] |  |  |

*Notes*: NMR, neonatal mortality rate; CI, confidence interval; Quin., Quintile. The small number of observations and possible non-linear relationships implies that the trend estimates should be treated with caution.
